# Successes and Lessons Learned in Moving All Education and Service Activities to Virtual Learning and Services

**DOI:** 10.1093/geroni/igab046.1832

**Published:** 2021-12-17

**Authors:** Sam Cotton, Anna Faul, Pamela Yankeelov, Barbara Gordon, Joseph D'Ambrosio

**Affiliations:** 1 University of Louisville Trager Institute, University of Louisville Trager Institute, Louisville KY, Kentucky, United States; 2 University of Louisville, University of Louisville, Kentucky, United States; 3 University of Louisville, Louisville, Kentucky, United States

## Abstract

Despite technology challenges, the UofL GWEP was able to provide access to quality care during the pandemic by pivoting all health and behavioral health patient appointments to telehealth. We found being flexible and using technology patients were most familiar with, was the most successful way easing into telehealth. We trained providers in a variety of technology tools and modalities to support this flexibility. We implemented a remote patient monitoring program and a virtual friendly visitors’ program for our most vulnerable patients. Our workforce development focus supported us in teaching our 60+ interdisciplinary interns how to conduct telehealth visits, how to collaborate as an interdisciplinary team in managing the remote patient monitoring program, and how to do virtual case conceptualization and care planning meetings. To re-create the dynamic atmosphere of an interprofessional learning experience, we have paired students together in teams, and provide them with regular opportunities to meet and engage.

